# Editorial: Astrocytes, a Kaleidoscope of Diversities, a Pharmacological Horizon

**DOI:** 10.3389/fphar.2021.638239

**Published:** 2021-03-08

**Authors:** Lorenzo Di Cesare Mannelli, Stefania Ceruti, Juan A. Orellana

**Affiliations:** ^1^ Department of Neuroscience, Psychology, Drug Research and Child Health – Neurofarba – Section of Pharmacology and Toxicology, Università degli Studi di Firenze, Florence, Italy; ^2^ Department of Pharmacological and Biomolecular Sciences, Università degli Studi di Milano, Milan, Italy; ^3^ Departamento de Neurología, Escuela de Medicina and Centro interdisciplinario de Neurociencias, Facultad de Medicina, Pontificia Universidad Católica de Chile, Santiago, Chile

**Keywords:** glia, neurorestoration, astrocyte population, astrocyte polarization, neuroplasticity, pharmacological targets

Astrocytes are specialized glia, vital for neural circuit function, and represent a population of complex and functionally diversified cells (Chai et al., 2017). Physiological multiplicity of astrocytes is apparent among different brain circuits and microcircuits, further individual astrocytes display heterogenous signaling properties depending on the subcellular compartments. With respect to injury and disease, astrocytes undergo several phenotypic changes that may be protective or deleterious with regard to pathology in a context-dependent manner (Liddelow and Barres, 2017). Damages to the peripheral and central nervous tissue as well as pathological alterations of complex organs, like the intestine, lead to astrocyte activation, causing neuroanatomical and neurochemical transformations which sustain pathological signals participating in maladaptive plasticity. Nevertheless, also during pathology, astrocytes (as a whole or specific phenotypes or some yet-to-be identified population) maintain their neuroconservative role (Zhou et al., 2020).

Thus research has the challenge to pharmacologically regulate astrocyte functions with special focus on reducing neural aberrant excitation and promoting restorative signals.

The present research topic is intended to be a collection of new physiological and pathological evidence regarding astrocyte features and functions focusing on the concept that astrocytes represent a highly variegated population of cells that mediate neural circuit-specific roles in health and disease.

Spampinato et al. have focused on two important astrocyte functions with pathophysiological relevance: i) regulation of neural stem cell properties within adult neurogenic niches, positive pleiotropic actions of utmost importance under neurodegenerative conditions as an attempt to replace lost cell populations and ii) regulation of the integrity and functions of the blood-brain barrier (BBB) in physiological condition and as a reaction to harmful events contributing to either exacerbate or reduce BBB damage.

Another crucial physiological need satisfied by astrocytes is the cleansing of the cerebral tissue from waste molecules. Aquaporin-4 (AQP-4), a brain water channel, plays a pivotal role in this process. As shown in the review article of Valenza et al., it is mainly expressed on astrocytic endfeet closest to blood vessels participating in several astrocyte signals. The review points out the latest AQP-4 findings related to aging and Alzheimer’s disease as well as the available knowledge on pharmacological tools to target AQP-4.

As regards the cross-talk with the other nervous cells, classic astrocyte-to-neuron communication encompasses the release of messengers via exocytosis, carrier membrane transport and opening of a wide-range of channels (Gundersen et al., 2015). Nevertheless, recent evidence indicates that brain cells may communicate via alternative pathways, including the release of exosomes (Frühbeis et al., 2013). In this context, Venturini et al. have found that astroglial processes could release neurogloblin-containing exosomes as new non-conventional signals.

Among channels implied in the intercellular crosstalk, connexins (Giaume et al., 2021) represents a conserved family of membrane proteins that allow the ionic and molecular exchange between the cytoplasm of adjacent cells (through gap junction channels) or the communication between the extracellular and intracellular space (via hemichannels) (Leybaert et al., 2017). On this subject, Lagos-Cabré et al. have reviewed and discussed evidence suggesting that cell adhesion and cytoskeletal dynamics, both of which are relevant to cell migration, take place by modulation of hemichannels rather than gap junction channels.

Despite the evident astrocyte complexity in terms of phenotype and function, the molecular basis of these differences are unclear. Lozzi and co-workers, by using bioinformatic approaches have demonstrated that cohorts of transcription factors may modulate region-specific molecular signatures in astrocytes. This evidence points out the idea that differential expression of transcription factors governs astrocyte diversity in the brain parenchyma.

Melatonin is produced in the pineal gland and released according to the circadian rhythm (Cipolla-Neto and Amaral, 2018). Recently, this hormone has received attention due to its neuroprotective effect via Nrf2 pathway (Cao et al., 2017). In this issue, Chen and co-workers showed the protective action of melatonin from heme-induced toxicity observed upon intracerebral hemorrhage. They found that this response is mediated by the activation of M2 receptors and the transcription factor Nrf2.

Astrocytes do not express endothelin-1 (ET-1) in healthy conditions, but they prominently express and release this protein in multiple sclerosis demyelinated plaques (D'haeseleer et al., 2013). In this scenario, the work of Hostenbach et al. determined that diversity of pro-inflammatory cytokines causes the production of ET-1, the latter being dramatically prevented by the statin and the natural phenol simvastatin and resveratrol, respectively.

The relevance of astrocytes in pathological conditions was deepened by Siracusa et al. The loss of astrocyte functionality as a result of cellular senescence has been related to neurodegenerative disorders as well as to aging. Astrocytes can drive the inflammatory response and contribute to the altered neuronal activity in several frontal cortex pathologies such as ischemic stroke and epilepsy. For these reasons, the authors discuss the possibilities to target astrocytes as an approach toward pharmacological therapies.

In this view, astroglia is implicated in the pharmacodynamic of already known products. Recent developments have demonstrated that astrocytes can indeed be the cellular targets of neuroprotective agents. As demonstrated in the paper by Zhao et al., vinpocetin, a semi-synthetic alkaloid from the leaves of *Phyllostachys pubescens*, has anti-inflammatory, anti-oxidant and anti-apoptotic actions both *in vitro* following oxygen-glucose deprivation and *in vivo* against ischemia/reperfusion injury by targeting specific astrocytic pathways. Specifically, it promotes Connexin43 phosphorylation through the PI3K/Akt pathway, which in turn promotes BBB integrity, cell-to-cell communication with an overall reduction in brain edema and tissue damage.

The natural compound 2,7,2′-trihydroxy-4,4′7′-trimethoxy-1,1′-biphenanthrene (TTB) isolated from the orchid *Liparis nervosa* (Thunb.) Lindl. has been studied by Liu et al. in an *in vitro* model of oxygen-glucose deprivation/reoxygenation injury (OGD/RI) on astrocytic cultures to mimic the pathological condition named neonatal hypoxic/ischemic. Data demonstrate that TTB is effective against cell death preserving the intracellular antioxidant activity by activating the transcription factor nuclear factor erythroid 2-related factor 2 (Nrf2) and related pathways. Additionally, TTB reverts neurite loss induced by OGD/RI in neuron-astrocyte cocultures.

In conclusion, this Research Topic offers novel information about the role of astrocytes in neurophysiology and in neuropathology as well as possible therapeutic approaches. The pharmacological modulation of astrocytic targets is encouraged as a breakthrough strategy for the relief from several debilitating pathologies.
